# Newborn intensive care survivors: a review and a plan for collaboration in Texas

**DOI:** 10.1186/s40748-015-0025-2

**Published:** 2015-10-19

**Authors:** Alice Gong, Yvette R. Johnson, Judith Livingston, Kathleen Matula, Andrea F. Duncan

**Affiliations:** Department of Pediatrics, The University of Texas Health Science Center at San Antonio, 7703 Floyd Curl Dr., San Antonio Texas, 78229 USA; Cook Children’s Hospital, 1500 Cooper St., Dodson Specialty Building, 2nd Floor, Fort Worth, TX 76104 USA; The University of Texas Health Science Center-Houston, 6431 Fannin St.,, Houston Texas, 77030 USA

**Keywords:** Preterm birth, Developmental follow-up, Standardized practice, Collaboration, Outcomes research

## Abstract

**Background:**

Neonatal intensive care is a remarkable success story with dramatic improvements in survival rates for preterm newborns. Significant efforts and resources are invested to improve mortality and morbidity but much remains to be learned about the short and long-term effects of neonatal intensive care unit (NICU) interventions. Published guidelines recommend that infants discharged from the NICU be in an organized follow-up program that tracks medical and neurodevelopmental outcomes. Yet, there are no standardized guidelines for provision of follow-up services for high-risk infants.

The National Institute of Child Health and Human Development Neonatal Research Network and the Vermont Oxford Network have made strides toward standardizing practices and conducting outcomes research, but only include a subset of developmental follow-up programs with a focus on extremely preterm infants. Several studies have been conducted to gain a better understanding of current practices in developmental follow-up. Some of the major themes in these studies are the lack of personnel and funding to provide comprehensive follow-up care; feeding difficulties as a primary issue for NICU survivors, families, and programs; wide variability in referral and follow-up care practices; and calls for standardized, systematic developmental surveillance to improve outcomes.

**Findings:**

We convened a one-day summit to discuss developmental follow-up practices in Texas involving four academic and three nonacademic centers. All seven centers described variable age and weight criteria for follow-up of NICU patients and a unique set of developmental practices, including duration of follow-up, types and timing of developmental assessments administered, education and communication with families and other health care providers, and referrals for services. Needs identified by the centers focused on two main themes: resources and comprehensive care. Participants identified key challenges for developmental follow-up, generated recommendations to address these challenges, and outlined components of a quality program.

**Conclusions:**

The long-term goal is to ensure that all children maximize their potential; a goal supported through quality, comprehensive developmental follow-up care and outcomes research to continuously improve evidence-based practices. We aim to contribute to this goal through a statewide working group collaborating on research to standardize practices and inform policies that truly benefit children and their families.

## Introduction

Neonatal intensive care is a remarkable success story. Survival rates of infants weighing <800 grams increased from 0 % in 1943–1945 to 34 % in 1987–1988 and 70 % in 1994 [1]. In the early 1980s, preterm newborns <28 weeks gestation had a 90 % mortality rate. Recently the Eunice Kennedy Shriver National Institute of Child Health and Development (NICHD) Neonatal Research Network (NRN) reported survival of 65 % and 56 % survival without severe impairment in infants <27 weeks gestation [2].

Extraordinary advances in obstetric and neonatal care have resulted in tremendous gains for the premature infant. The efforts and resources invested to achieve such gains come at a significant cost. The financial cost alone of neonatal intensive care has been estimated at $3,400 per hospital day [3]. Preterm births cost the U.S. health care system more than $26.2 billion in 2005 [4].

The substantial investment in neonatal care has resulted in improved mortality and morbidity outcomes of preterm infants. However, the significant decrease in mortality and short-term morbidities has not had a proportionate effect on long-term neurodevelopmental outcomes. Rigorous long-term outcome studies evaluating the impact of neonatal care and perinatal interventions have informed our understanding of their impact on long-term outcomes and helped to refine the care that has resulted in improved outcomes. Research on perinatal interventions, such as studies supported by NICHD, demonstrate the impact of obstetric and neonatal care on long-term outcomes of neonatal intensive care unit (NICU) survivors. Several key examples include findings that show: (1) Apgar scores do not predict cerebral palsy [5]; (2) the clear benefits of antenatal corticosteroids in reducing the risk of life-threatening morbidities including respiratory distress syndrome and intraventricular hemorrhage (IVH), balanced against studies demonstrating lack of benefit and possible harm from repeated courses [6]; (3) lack of benefit of antenatal treatment with magnesium sulfate on school-age outcomes [7]; (4) improved childhood outcomes following hypothermia for neonatal encephalopathy [8] and (5) benefit of early caffeine administration among preterm infants <1250 grams at birth and reduction of bronchopulmonary dysplasia (BPD) and improved long-term developmental outcomes [9].

The value of follow-up is also emphasized by studies isolating factors that impact long-term outcomes. For example, certain morbidities (e.g. IVH, necrotizing enterocolitis [NEC], BPD, retinopathy of prematurity [ROP]) have been identified that adversely affect outcomes whereas certain psychosocial factors (e.g. higher maternal and paternal IQ and socioeconomic status) positively impact child developmental outcomes and loss to follow-up [10–14]. Guillén, et al. [15] conducted a systematic review to assess center variation in rates of neurodevelopmental impairment (NDI) at 18–24 months corrected age among extremely low-birth-weight (ELBW) or extremely low-gestational-age (ELGA) infants. NDI was defined as the presence of at least one of the following: a mental developmental index score two standard deviations below the mean on the Bayley Scales of Infant Development-Second Edition®; cerebral palsy (nonprogressive motor impairment characterized by abnormal muscle tone in at least one extremity and a decreased range or control of movements); visual impairment (visual acuity of less than 20/200 unilaterally or bilaterally or blindness); or significant hearing impairment (hearing loss requiring amplification). NDI and follow-up rates were reported for 34,185 infants from 20 publications involving 24 cohorts. Follow-up rates ranged from 71–100 % and higher rates of NDI correlated with loss to follow-up rates, suggesting the healthier children may not be followed [15].

Van der Pal-de Bruin et al. [16] reported on a Dutch cohort of 1,338 infants born <32 weeks gestation or very low birth weight (<1500 grams; VLBW) for whom data was longitudinally collected from birth through age 19 years. Follow-up data were captured for 74 % participants still alive at age 19 years. Outcomes assessed included physical, cognitive, behavioral, quality of life measures, and impact of disabilities. Study participants demonstrated significant developmental impairment over time. However, major disabilities were unchanged as children aged but minor disabilities increased [16]. A German study of 148 children with birth weights <1000 grams analyzed the relationship between perinatal risk factors, social parameters, and neurodevelopmental outcomes at 10–13 years. Results indicated that regardless of brain compromises, neurodevelopmental outcomes between 10–13 years of age were better among children from more educated mothers: low maternal education was the strongest factor associated with a decreased composite intelligence quotient (IQ) [14].

The NICHD has recommended follow-up for all ELBW infants to assess growth, neurologic status, behavior, language, socioeconomic status, and family resources [17]. The American Academy of Pediatrics (AAP) Committee on The Fetus and Newborn’s 2008 guidelines for hospital discharge of high risk neonates, state that “most high-risk infants should be enrolled in a follow-up clinic that specializes in the neurodevelopmental assessment of high-risk infants” and that “standardized assessments should be performed in the follow-up clinic at specific ages through early childhood” [18]. Furthermore, guidelines published by the AAP and American College of Obstetrics and Gynecology [19] recommend that infants discharged from the NICU be in an organized follow-up program that tracks and records medical and neurodevelopmental outcomes for analysis later; this follow-up is an essential component of level 3 and level 4 services. Data from multiple follow-up studies indicate that perinatal therapeutic interventions may dramatically alter later growth and development and there is increased recognition of the potential disconnect between perinatal and long-term outcomes [1, 17]. Yet there are no standardized guidelines for provision of follow-up services for high-risk infants and inadequate support for follow-up programs [18, 20]. However, there are initiatives designed to reach this goal such as the MedImmune and National Initiative for Children’s Healthcare Quality’s toolkit for follow-up of premature infants up to 12 months chronological age [21].

## Review of practices in developmental follow-up

Several U.S. studies have been conducted to gain a better understanding of current practices in developmental follow-up, including online surveys of follow-up programs associated with academic and private practice based follow-up programs [20, 22]; retrospective analysis of referral data from California Children’s Services [23]; and analysis of programs associated with the NICHD NRN [1]. Follow-up practices in states such as Rhode Island [24], as well as in other countries, such as Australia [25, 26] and Canada [27-30], have also been described. Some of the major cross-cutting themes in these studies are the lack of personnel and funding to provide comprehensive follow-up care, the significant feeding difficulties among NICU survivors, wide variability in referral and follow-up practices, and calls for standardized, systematic developmental surveillance to improve outcomes.

The need for developmental follow-up and outcome studies is recognized by leaders and organizations concerned about the impact of preterm birth. The NICHD NRN and Vermont Oxford Network (VON), a non-profit organization that maintains a database on medical interventions and outcomes for preterm infants at member institutions worldwide [31], are working toward standardizing practices and conducting outcomes research. However, these organizations only include a subset of developmental follow-up programs.

We, too, are concerned about the need for standardized practices and outcomes research and sought to have a better understanding of perspectives on developmental follow-up practices in Texas through a focused dialogue with stakeholders. Texas is a large state with multiple academic and private developmental follow-up programs. In 2012, the total number of live births in Texas was 382,438 and 8.3 % of newborns were low birth weight (<2500 grams) [32]. Among Texas mothers, 42.3 % were unmarried, only 62.6 % received prenatal care in the first trimester of pregnancy with 53.8 % of births covered by Medicaid [32]. In 2013, the preterm birth rate in Texas was 12.3 % (compared with the U.S. rate of 9.6 %), with higher rates for Blacks and Hispanics compared to Whites [33]. Sixteen percent of Texas women, ages 18–24, had less than a high school education as compared to 13 % of U.S. women. For women ages 25 and over, 18.3 % as compared to 13.4 % of U.S. women did not have a high school diploma [34]. These data underscore the many challenges to ensuring a healthy start for Texas newborns, particularly those born premature.

Developmental follow-up of high-risk infants in Texas exemplifies the problems nationally as they relate to socioeconomic factors that impact developmental and behavioral outcomes. These risk factors include maternal education, adolescent pregnancy, poverty, and race/ethnicity. Hoffman et al. [35] found that premature infants born to adolescent mothers, who are less educated, single, and of Hispanic or African American race/ethnicity are at significantly higher risk for adverse behavioral outcomes and cognitive impairment at 18–22 months corrected age. Using the Bayley Scales of Infant and Toddler Development, Third Edition® (BSITD III), premature infants of adolescent mothers have lower composite language scores of <85 (56 % vs. 49 %, *p* = 0.07). These infants are also significantly more likely to have unstable housing, be under state supervision, and have multiple rehospitalizations compared to similar premature infants born to older, better-educated mothers. Using the Brief Infant Social Emotional Assessment (BITSEA), premature infants of adolescent mothers had higher scores of behavioral and social problems compared to premature infants of adult mothers (mean 14.8 vs. 12.1, *p* < 0.001). These findings underscore the need to include premature infants and their families in a comprehensive follow-up program that includes care coordination and education to ensure optimal outcomes [35].

## Findings

### First annual summit on follow-up practices in Texas

On February 20, 2015, we convened a one-day summit to discuss follow-up practices in Texas among centers offering care for children and families affected by preterm birth. Our objective was to initiate dialogue about NICU follow-up best practices and explore the feasibility of creating a working group focused on long-term follow-up of high-risk NICU survivors. An email invitation was sent to nine Texas follow-up programs and, of these, four academic and three non-academic programs participated. Representatives from the NICHD, Texas Department of Assistive and Rehabilitative Services’ Early Childhood Intervention (ECI) program, and Hand to Hold, a family advocacy group for NICU survivors, also participated in the meeting.

Prior to the meeting, follow-up program directors were asked to present information about the following: (1) patient population; (2) a list of personnel who perform developmental, behavioral, and psychological testing; (3) follow-up practices; (4) the names of developmental tests administered; (5) the age and schedule of testing; (6) the processes for reporting results; (7) the procedure for referrals and interventions; (8) the duration of tracking; (9) the interface with neonatology and primary care colleagues; (10) the experience with impact of follow-up; (11) the perceived needs of follow-up practice; and (12) a perspective on feasibility of a statewide collaboration.

## Center reports

Across the seven centers, there was significant variability in follow-up practices (Table 1), although not all reported on every item listed above. Age and weight criteria for NICU patients were different at all centers as was the annual census. However, a common feature among centers was that they operated with a small, part-time staff. All seven of the centers described a unique set of developmental practices, including duration of follow-up, types and timing of developmental assessments administered, education and communication with families and other health care providers, and referrals for subspecialty services. Despite the variation in assessments performed, all centers used the BSITD-III at some point. Only two of the centers administered the scales at the same age (18–24 months post-menstrual age) and one of these two centers outsourced the testing separate from the clinic visit while the other administered the scales at the time of the clinic visit. Most centers are correcting for gestational age up until age two; this process is generally well accepted [17]. Due to increased survival of the most immature and fragile infants, centers discussed whether two years of correcting for gestational age is still appropriate; however there are no data to guide a change in practice.

**Table 1:** Select data from seven NEON follow-up centers

Patient population	2 centers ≤ 1500 g or < 32 weeks
	1 center ≤ 1500 g or ≤ 32 weeks
	1 center < 1500 g
	1 center < 800 g
	1 center < 27 weeks, in any research study or other at team discretion
	1 center – extended list of NICU graduates
	Annual census ranged from 200 (private center) to 5,200 (private center with three hospital systems)
	84 % was highest percentage of Medicaid NICU patients
Personnel	3 academic and 1 private center were supervised by neonatologists; 1 academic and 2 private centers were supervised by developmental pediatricians
	All 7 centers had small, often part-time staff
	5 centers “borrowed” staff from hospital NICU
	3 academic centers used physicians-in-training
	Types of non-physician professional staff involved in follow-up included: advanced nurse practitioner, nurse, social worker, physical therapist, occupational therapist, speech therapist (needed most for feeding therapy), audiologist, dietician, nutritionist, lactation consultant, psychometrician, and case manager.
Duration of follow-up	2 years up to 22 years, although the majority of centers reported following patients for 5 years or less.
Communications with families	2 centers reported providing verbal and written feedback to parents about testing at the time of the visit
	4 centers reported providing feedback to the parents at the time of the visit, as well as mailing written reports to parents and primary care providers after clinic visits
	1 center did not include communications in the report
Communications with providers	1 academic center and 2 private centers reported providing updates and maintaining on-going communications with their neonatology groups. The academic center and one of the two private centers reported trying to engage with community pediatricians and include them in meetings or on a committee, while the other private center reported functioning as a de facto medical home. A third private center commented, “We let the pediatricians drive.”

General comments about the impact of follow-up were that it allows care coordination and continuity with the NICU and helps eliminate fragmentation in systems of care. Follow-up facilitates early identification of growth and feeding problems and earlier referrals to subspecialty care and rehabilitative services. Earlier referral helps to ensure children receive needed subspecialty medical care and frequent and longitudinal provision of information to families about their children’s developmental status. This aids in the prevention of developmental delay and secondary social, emotional, or behavioral problems. Ultimately, follow-up improves developmental outcomes. One optimal goal articulated was to provide follow-up through school age to assess and improve school readiness and school performance.

Needs identified by the centers focused on two main themes: resources and comprehensive multidisciplinary care. In terms of resources, centers reported needing funding to support infrastructure and research. Adequate resources would support the centers’ vision to provide comprehensive acute and well-baby care at least through transition to primary care providers. A comprehensive care follow-up program would adhere to and effectively implement established best practice guidelines (Table 2).

**Table 2:** Components of a quality comprehensive care NICU follow-up program

Personnel	• A multidisciplinary team with adequate staffing from physicians, psychologists, nurses, social workers, physical, occupational, speech, and respiratory therapists, nutritionists, lactation consultants, case managers, and ECI collaborators
	• Support for case management and home visits
Practices	• A standardized manual of operations
	• Processes to engage effectively with neonatologists, community pediatricians, and other primary care providers including data sharing linkages
	• Mechanisms for tracking during and after clinic discharge, including follow-up at school age, adolescence, and adulthood
	• Databases for tracking and research
Programs	• Family support groups
	• Organized educational program for outreach to families, providers, and community
	• Website with resources for families, providers, and community
Facilities	• Appropriate clinic space

### Key follow-up challenges identified

Summit participants discussed a number of systemic challenges for NICU survivors. Among these, length of stay (LOS) with urgency to discharge babies from the NICU is a major issue. Hospitals, insurers, families, and society may be unaware that an adequate support structure both during the NICU stay and during transition home is vital to ensure safe discharge.

Feeding challenges in the NICU have a major impact on LOS. NICU discharges are frequently focused on infant weight gain and achievement of full oral feedings that are not always sustained after discharge. This issue becomes a major problem for NICU survivors and their families as ability to feed the infant then becomes stressful. It is well recognized that breast milk is the best source of nutrition, providing protective immunities, growth hormones, and other elements tailored to the newborn’s needs [36, 37]. Low birth weight infants fed predominately with mothers’ own milk had better outcomes and less viral infections up to 8 months of life [38]. Exclusive breastfeeding is ideal; however, this presents many challenges for VLBW infants and their mothers [39]. NICUs need to change many practices in order to help families achieve optimal feeding goals after discharge. Many mothers forego their desire to breastfeed in order to get the baby home sooner since bottle-feeding is perceived as easier and faster [40]. Frequent post-discharge feeding issues include: mothers unable to breastfeed directly and resorting to continued pumping and feeding from bottles; babies not thriving, development of oral aversion, and feeding refusal; primary care providers changing to formulas that are inadequate for the growing premature infant or difficult to access in the outpatient setting and adding medications inconsistent with current guidelines.

All NICU survivors are at risk for neurodevelopmental deficits but, due to resource limitations, follow-up clinics are usually reserved for those who are sickest and have the most need. Some clinics are open to all who seek services but indigent populations, for a variety of reasons, often do not present to these clinics. The challenge for most developmental follow-up programs is ensuring long-term follow-up of all who are at risk, not just the sickest premature infants. Organized follow-up of all at-risk children is extremely important, as preterm children remain at risk for severe behavioral and cognitive deficits at school-age, even in the absence of early global deficits [41] and more subtle harbingers of these later deficits may present in very early life [42]. Lack of organized follow-up of all at-risk children and the resulting delay in early recognition of abnormalities may result in a missed opportunity for interventions aimed at improving modifiable outcomes. Limited budgets and resources encountered by many follow-up programs present a significant challenge to casting “a wider net” to include a representative population of premature infants at risk.

Families often face barriers to receiving follow-up services. Delays in first follow-up appointments because of staffing shortages or other systemic problems impact families’ access and use of follow-up services. When a follow-up program is funded exclusively through patient revenue, it negatively impacts follow-up rates [20]. Lack of insurance or inadequate insurance coverage and high co-pays for mental health services prevent children from receiving neurodevelopmental testing. From the parent’s perspective, one of the greatest values of developmental testing is the opportunity to learn about growth and development and tools and strategies to aid their child’s development. Parents of all socioeconomic levels are capable of learning to interact responsively with their children. Trials of parenting interventions in early childhood have demonstrated improvement in behavioral problems and responsivity [43]. Further, in a study of an intervention aimed at improving parental responsivity, VLBW children showed greater gains after the intervention than their term-born counterparts [44]. Given the tools and education, they can do the work to preserve and protect the care initiated in the NICU to help their children survive and thrive.

Follow-up programs are charged with identifying the need for therapy services among a population at high risk for delay. The tools used by most programs are not screeners; they are full assessments that identify delays warranting intervention. Screening has little purpose in an inherently high-risk population and is not effective in identifying subtle impairments [45]. Screening instruments are likely to under-identify infants in need of services, in part because they rely on parent report. Parental report may be unreliable if the questions are misunderstood or the parent has not attended to the child’s skills in all areas of development. One major challenge when referring a child to ECI is that Texas ECI programs are mandated to use only a customized version of the Battelle Developmental Inventory™ [46], and cannot accept results from any other developmental assessment. In many instances, assessments offered by follow-up programs should be accepted because these are based upon lengthy administrations of standardized clinical assessments, along with neuromotor exams provided by highly qualified professionals. This would save the ECI programs time and expense of performing a redundant service. Texas ECI is underfunded with an annual allocation of $400 per child as of February 2015. To finance operations, ECI is currently billing Medicaid and managed care organizations.

## Recommendations for statewide collaboration

Two overarching recommendations were made at the meeting as steps toward achieving quality comprehensive follow-up care in Texas: (1) create a statewide database and (2) establish uniform, evidence-based guidelines for developmental follow-up. Consensus should be reached about which populations, outcome variables, and long-term outcomes to track, as well as duration of follow-up. Data could also be used for benchmarking and linking immediate and long-term outcomes, collaborative statewide quality improvement initiatives, and standardizing protocols for ECI and Women, Infant, and Children (WIC) programs. Perhaps most importantly, levels of care for follow-up need to be established as not all NICU survivors will need every test and service.

A proposal was made to create a database and establish guidelines through the formation of a statewide consortium of high-risk providers to share data on best practices, outcomes, and protocols. The consortium could also partner with the VON, Texas Pediatric Society, Texas Association for Infant Mental Health, Texas Society for Clinical Social Work, and other groups. To this end and in deliberations after the February meeting, the group named itself the Neonatal Evaluation and Outcomes Network (NEON) and began the process to develop infrastructure to function. Given the current milieu and challenges in follow-up, the meeting participants outlined a series of recommendations that might be pursued by the network, organized thematically in three areas (Table 3).

**Table 3:** NEON recommendations for achieving quality comprehensive follow-up care

Systems	• Develop guidelines to determine levels of post-NICU discharge care based upon current knowledge and to update levels as data is acquired.
	• Start a database with meaningful, de-identified data that can be shared.
	• Choose common data points to gather from all units and build incrementally.
	• Educate hospitals, insurers, and society that time in the NICU is treatment as maturity is important for survival.
	• Work toward establishing universal nutrition guidelines for NICU and beyond.
	• Build consensus that breast milk is best.
	• Develop more family friendly NICUs.
	• Gather data to support that formula-fed premature infants need post-discharge preterm formula for the length of time determined by the medical specialist following the child.
	• Engage state and national professional organizations to promote support for quality comprehensive follow-up care, including advocacy to ensure ECI has adequate resources to provide timely and appropriate early intervention services.
	• Advocate at the state level for ECI acceptance of a referral for services based upon a comprehensive developmental evaluation by an appropriate professional.
	• Educate policy makers and insurers to change the culture away from waiting for a problem to occur to a prevention orientation, especially in vulnerable populations.
	• Educate WIC on post-menstrual age versus chronological age and dietary issues.
Families	• Provide support to families by focusing on children’s progress and what families are doing well, rather than just their deficits.
	• Empower parents.
	• Give educational information in multiple modalities addressing the needs of the adult learner (e.g. web-based resources, handouts, information videos, “just-in-time” educational or interactive tools). Information should be varied and repetitive to enhance learning and overall impact.
	• Help parents learn how to engage with health care professionals.
	• Provide education that time in the NICU is a treatment and impacts brain development.
	• Facilitate appropriate discharge with training and preparation for parents.
	• Provide education to families on how to breastfeed and use a breast pump and ensure the best kind of pump is immediately available.
	• For formula-fed infants, ensure families know how to mix formula correctly.
	• Arrange initial follow-up visits (and link with ECI) before NICU discharge.
	• Continue Post-discharge support with outpatient care, home visits, and phone calls.
	• Set up Life Line for families to call for guidance and assistance finding resources and family support groups such as Hand to Hold.
	• Create a Text for Baby for preterm babies modeled on the program for term babies.
	• Keep in mind what matters to families, e.g. will my child go to kindergarten with the rest of the children?
	• Teach parents about the role of early intervention to decrease the stigma.
Providers	• Educate neonatologists about why follow-up is part of the NICU continuum of care.
	• Educate community pediatricians that follow-up supports, not supplants, their work.
	• Educate community pediatricians caring for NICU survivors about existing guidelines.
	• Educate providers to help ensure they are helping families use evidence-based, developmentally appropriate feeding practices.

## Discussion

Many NICU graduates have survived multi-organ disease such as IVH, BPD, ROP, and NEC and have life-long complications from them. We frequently send them out of the NICU to be cared for by overwhelmed families and over-burdened primary care providers who do not have the time to provide a proper medical home. As we learn more about the devastating changes happening to fragile children during a period of great vulnerability in the NICU, we need to continue to research the consequences of care and how to help these children develop to their full potential.

There are many unanswered questions. As scientists, are we doing our due diligence if we are not tracking the outcomes of NICU survivors? What are the long-term consequences of NICU interventions? What are the unintended consequences of interventions on NICU survivors and their families? What are the factors that affect outcomes and how do these impact disease mechanisms and processes of resiliency? In Texas, are there disparities in referral, based on maternal race or ethnicity, similar to what was reported in California [23]? If so, what are the causes and how can these be addressed?

The Colorado Department of Public Health and Environment convened a stakeholder summit in 2012 to "optimize the health and developmental outcomes of premature and high risk infants and their families by sharing best practices and systems of care that support the transition home from the NICU and hospital to the medical home and supportive community-based services" [47]. The multidisciplinary groups participating came to many of the same conclusions as the Texas summit. With the growing awareness of the needs of the NICU survivor, it is time to link collaboratives and develop national databases in order to answer some of the key questions.

## Conclusion

“*I am delighted to approve the legislation authorizing the creation of a National Institute of Child Health and Human Development*. . . . *The future health of our Nation rests on the care of our children and the development of our knowledge of the medical and biological sciences*. . . . *Research in recent years has established beyond question that adult behavior, intelligence, and motivation are established by the experience and patterns of response developed in the formative years of life*. . .”

President John F. Kennedy, signing HR 11099, Public Law 87–838 (76 Stat. 1072), on October 17, 1962 [48].

The long-term goal of the NEON group is to ensure that all Texas infants reach their full potential. This goal is supported through quality, comprehensive developmental follow-up care and outcomes research to continuously improve evidence-based practices. We aim to reach this goal through collaboration and research for practice standardization and policy information that truly benefits Texas children and their families.

## Abbreviations

AAP: American Academy of Pediatrics
BITSEA: Brief Infant Social Emotional Assessment
BPD: Bronchopulmonary Dysplasia
BSITD: Bayley Scales of Infant and Toddler Development
ECI: Early Childhood Intervention
ELBW: Extremely Low-Birth-Weight
ELGA: Extremely Low-Gestational-Age
IQ: Intelligence Quotient
IVH: Intraventricular Hemorrhage
LOS: Length of Stay
NDI: Neurodevelopmental Impairment
NEC: Necrotizing
NEON: Neonatal Evaluation and Outcomes Network
NICHD: National Institutes of Child Health and Development
NICU: Neonatal Intensive Care Unit
NRN: Neonatal Research Network
ROP: Retinopathy of Prematurity
VLBW: Very Low Birth Weight
VON: Vermont Oxford Network
WIC: Women, Infants, and Children
